# Depressive symptoms in people with chronic physical conditions: prevalence and risk factors in a Hong Kong community sample

**DOI:** 10.1186/1471-244X-12-198

**Published:** 2012-11-14

**Authors:** Hairong Nan, Paul H Lee, Ian McDowell, Michael Y Ni, Sunita M Stewart, Tai Hing Lam

**Affiliations:** 1 Department of Community Medicine, School of Public Health, The University of Hong Kong, Unit 624-627, Level 6, Core F, Cyberport 3, 100 Cyberport Road, Hong Kong SAR, China; 2 Department of Epidemiology and Community Medicine, University of Ottawa, Ottawa, Canada; 3 Department of Psychiatry, University of Texas Southwestern Medical Center at Dallas, Dallas, USA

**Keywords:** Depressive symptoms, Chronic conditions, Family functioning, Chinese, PHQ-9, Community, Populations

## Abstract

**Background:**

Depression is predicted to become one of the two most burdensome diseases worldwide by 2020 and is common in people with chronic physical conditions. However, depression is relatively uncommon in Asia. Family support is an important Asian cultural value that we hypothesized could protect people with chronic physical conditions from developing depression. We investigated depressive symptom prevalence and risk factors in a Chinese sample with chronic medical conditions, focusing on the possible protective role of family relationships.

**Methods:**

Data were obtained from the Hong Kong Jockey Club FAMILY Project cohort study in 2009–2011, which included 6,195 participants (age ≥15) with self-reported chronic conditions. Depressive symptoms were recorded using the Patient Health Questionnaire-9 (PHQ-9). Demographic and lifestyle variables, stressful life events, perceived family support and neighborhood cohesion were assessed. Factors associated with a non-somatic (PHQ-6) depression score were also examined.

**Results:**

The prevalence of depressive symptoms (PHQ-9 scores ≥5) was 17% in those with one or more chronic conditions, and was more prevalent in women than in men (19.7% *vs*. 13.9%; *p* < 0.001). In multilevel analyses, life stress, number of chronic conditions and satisfaction with family support explained 43% of the variance in PHQ-9 scores (standardized regression coefficients of 0.46, 0.15, and −0.12 respectively, all *p* <0.001). Body mass index, problem alcohol drinking, physical activity, and unmarried status were significantly associated with PHQ-9 scores, although these associations were weak. Variables associated with depression explained 35% of the variance in non-somatic (PHQ-6) depression scores. Satisfaction with family support played a stronger protective role against depressive symptoms (both PHQ-9 and PHQ-6 scores) among women than men (*p* < 0.05).

**Conclusions:**

Acute life stress and the number of chronic conditions, together with socio-demographic factors, explain most variance in depressive symptoms among chronically ill Chinese individuals. Somatic items in the PHQ-9 increased the depression scores but they did not alter the pattern of predictors. Family support appears to be an important protective factor in Chinese cultures for individuals with chronic conditions.

## Background

Chronic physical conditions have been associated with an increased risk for depression in a range of cultural settings [1-4], and the risk has been shown to increase with the number of conditions [5]. Mainland China, Hong Kong and Taiwan have been undergoing rapid urbanization and Westernization of lifestyles and these changes are associated with increasing risk of chronic disease [6-8]. Nonetheless, Asian rates of depression remain relatively low. One estimate placed the prevalence of lifetime major depressive disorder in mainland Chinese communities at 6.1%, compared to 16.2% in the United States [9,10].

The co-occurrence of chronic conditions and depressive symptoms can increase suffering among patients and their family members. Yet few studies have reported on depression prevalence or risk factors in a large general population sample of people with chronic medical conditions. To further examine the relationship between chronic medical conditions and depression, this study examined the prevalence of depressive symptoms among people with chronic conditions in a large Chinese community sample in Hong Kong. The second objective was to analyze factors that predict the development of depressive symptoms among those with chronic conditions.

Risk factors for developing depression have been mostly described in studies in Western countries. Yet Eastern cultural values and practices may have a protective role in mental health, so cultural factors that could modify the association between chronic conditions and depression have been studied in chronically ill Chinese samples. However, previous studies were small and focused on particular groups such as the elderly [3,11], or were limited to telephone interviews [2]. Various factors may protect against the development of depression, some of which could explain the apparent low incidence of secondary depression in Asian populations. Social support mitigates the development of depressive symptoms in people with chronic conditions both in the West [12] and in China [13]. Indeed, social relations are of particular importance in Chinese populations [14], and family support plays an essential role in Eastern cultures influenced by Confucianism, particularly in times of illness [15] and during treatment [14]. An epidemiological survey in mainland China reported that depressive symptoms were more common in women than men [9]; a second study found that family support and health status explained most of the variation in depressive symptoms [15]. In studies from the West, lower social support is likewise associated with worse health status and more depressive symptoms over the year following an acute myocardial infarction, particularly for women [16]. Older women are more likely than older men to be adversely affected and to develop depressive symptoms when family support is weak [17]. Gender roles in Chinese societies are more differentiated than they are in the West [18], but a possible interaction between gender and family support in protecting against depressive symptoms in chronic illness has not been investigated. Hence, we examined the relationship between satisfaction with family relationships and depressive symptoms, and assessed whether this effect differed for women and men. Prior studies [16,17] have often analyzed social relationships at one level instead of examining them at multiple levels (family, work, or neighborhood) [19,20]. Support at each of these levels could confer protection against depressive symptoms so we examined this possibility using multi-level analyses.

Life stress has been shown to be associated with both psychological and physical problems [21], mostly in studies conducted in Western populations [22,23]. Recently, a case-control study from mainland China also found that patients with major depressive disorders were more likely to have experienced stressful life events than controls, and reported a dose-response relationship between stressful life events and major depressive disorders [24]. Therefore, an assessment of stressful life events has been included in the present study.

A potential complicating factor in studying depression in Asian populations is the tendency among some ethnic groups, such as Indians, Vietnamese, and Chinese, to somaticize their emotional experience [25,26]. For example, people in North India were found to describe depression using somatic metaphors such as ‘pressure on the mind’ or ‘sinking heart’ [25]. Such presentations could lead to depression being mislabeled as a chronic physical ailment. In East-Asian populations, the influence of Chinese cultural ideologies of emotional calmness, self-restraint and adaptive strategies reduces the acceptability of expressing affective symptoms and also blurs boundaries between physical and mental illness [27]. Compared with the Caucasian population, somatic symptoms may be a more common reason for Chinese adults to seek psychiatric services for their mental health problems [28]. In a community sample of elderly people in Hong Kong, depression showed an independent relationship with six medically unexplained somatic symptoms including loss of appetite, fatigue, insomnia, recurrent gastro-intestinal problems, recurrent headache and dizziness, after adjustment for social-demographic and medical conditions [29]. Therefore, we have compared the somatic and non-somatic symptoms of depression among individuals with chronic conditions.

The objectives of this study were 1) to investigate the prevalence of self-reported depressive symptoms in a large community sample of individuals who reported chronic conditions; 2) to analyze risk factors for depressive symptoms in this sample; 3) to examine the potential effects of life stress and family support on depressive symptoms; 4) to compare the somatic and non-somatic symptoms of depression among individuals with chronic conditions.

## Methods

### Study sample

A population-based household survey entitled ‘The FAMILY: a Jockey Club Initiative for a Harmonious Society’, was carried out from March 2009 to April 2011. Sampling was based on residential addresses provided by the Hong Kong Census and Statistics Department. First, residential addresses were randomly sampled in all of the 18 districts of Hong Kong, with sample sizes proportionate to the district populations. The random sampling was stratified by type of dwelling (e.g., public or private housing complexes). Before the survey began, a notification letter was sent to the selected households, explaining the purpose of the survey and assuring confidentiality. Trained interviewers then approached all selected residential addresses. We excluded those addresses in which the interviewers could not contact anyone after six visits, each at least one week apart. Each family member aged ≥15 years in the sampled household who could understand and respond to the interview in Cantonese was eligible and invited to participate. Family members were interviewed separately and privately, with data gathered in electronic format on laptop computers. Participants responded to a structured questionnaire under the supervision of the interviewer who was available to answer questions and clarify items. We only retained households in which all family members agreed to participate. Written consent was obtained and the study was approved by the Institutional Review Board of the University of Hong Kong.

### Health assessments

#### Chronic conditions

Respondents were asked whether they had any of the following chronic conditions, as diagnosed by a physician: hypertension, heart disease, stroke, diabetes, dyslipidemia, asthma, chronic obstructive lung disease, digestive diseases (gastric ulcer, hepatitis B or C, or cirrhosis), chronic musculoskeletal conditions (arthritis, rheumatism, low back pain, gout, and osteoporosis) or cancer. This list was derived from a panel of experts’ knowledge of the common medical conditions in Hong Kong. A similar list had been used in the Hong Kong Population Health Survey in 2003–2004 and in the Hong Kong Thematic Household Survey of 2009–2010. A simple count of the number of conditions was used in the present analysis.

#### Psychological assessments

The nine-item Patient Health Questionnaire (PHQ-9) [30,31] was used to record depressive symptoms. Respondents rated the frequency of experiencing nine symptoms during the previous two weeks: 0) not at all, 1) on several days, 2) on more than half of the days, and 3) nearly every day (see Appendix 1). Scores range from 0 to 27, with higher scores indicating more symptoms. For descriptive analyses, these scores were classified as indicating minimal depression (scores 0 to 4), mild (5 to 9), moderate (10 to 14), moderately severe (15 to 19) and severe depression (20 and over). We previously reported on the reliability and validity of the Chinese PHQ-9 using data from the FAMILY project [32]. The alpha internal consistency in those with chronic conditions was 0.83. Three items in the PHQ-9 refer to somatic symptoms (item 3, 4 and 5 in Appendix 1). These items could falsely identify depressive symptoms if they were applied to people with chronic health conditions, so we created a separate depression score omitting the somatic items, labeled PHQ-6 in the tables. We also report results for the somatic items separately, labeled PHQ-3 in the tables.

### Predictor variables

We tested predictors shown relevant in previous studies: age [33], gender [9], socioeconomic status [15], marital status [4], whether living alone or not [34]. Socioeconomic status was measured separately via education and family income, with education classified into three levels by the highest level attained: primary, secondary or tertiary. Monthly family income was classified into five levels: <5,000, 5,000 – 9,999, 10,000 – 19,999, 20,000 – 29, 999, and ≥30,000 Hong Kong dollars (US$ 1 = HK$7.8). Lifestyle variables included smoking [35], drinking [36], and physical activity [37,38]) and life stress [39]. Physical activity was recorded using by the International Physical Activity Questionnaire (IPAQ), classified into less active versus more active [40]. Smoking was classified into current smoker (at least one cigarette every day) versus current non-smoker. Drinking was classified into high risk drinking (5 or more glasses of alcohol on a single occasion, or drinking ≥ 210g (men) or ≥ 140g (women) of alcohol per week) versus low risk drinking (including non-drinkers).

We then focused on indicators of social support (satisfaction with family support [15], neighborhood cohesion [4]) as possible mitigating factors for depressive symptoms. Given the importance of family in Chinese culture [41], satisfaction with family support was hypothesized to be a protective factor for depression in Chinese with chronic conditions. Satisfaction with family supportiveness was measured using the 5-item Family APGAR score. This covers Adaptation (“I am satisfied that I can turn to my family for help when something is troubling me”), Partnership (“I am satisfied with the way my family talks over things with me and shares problems with me”), Growth (“I am satisfied that my family accepts and supports my wishes to take on new activities or directions”), Affection (“I am satisfied with the way my family expresses affection, and responds to my emotions, such as anger, sorrow, or love”), and Resolve (“I am satisfied with the way my family and I share time together”) [42]. Responses are Hardly ever (score 0), Some of the time (1), Almost always (2). Scores are summed, giving a range from 0 to 10, with higher scores indicating greater satisfaction with family support. The Chinese version has been used previously with some evidence of reliability and construct validity in Hong Kong [43]. The alpha internal consistency in those with chronic conditions in the FAMILY project was 0.94.

Perceived neighborhood cohesion was measured using 4 items, each rated on a 5-point Likert agreement scale: 1) “people around here are willing to help their neighbors”, 2) “this is a close-knit neighborhood”, 3) “people in this neighborhood can be trusted”, and 4) “people in this neighborhood do not get along with each other” (reverse coded) [44]. We omitted a fifth item (“people in this neighborhood do not share the same values”) from the original list because of its poor factor loading (<0.40). The total score was rescaled to run from 1 to 5, with higher scores indicating greater social cohesion. The original validation was based on a 1995 survey in Chicago, Illinois [44] and the four items have been shown reliable in three subsequent US populations (Baltimore, Forsyth County, and New York) [45]. These items had an alpha internal consistency of 0.86 in the present sample, but the neighborhood cohesion scale has not been formally validated in Hong Kong.

A 19-item stressful life event questionnaire, derived from the Recent Life Changes Questionnaire [46], included death of a family member or close friend, unemployment, relocation, and serious health, financial or interpersonal problems among others. Participants indicated whether any of these had occurred and if so, rated its impact on a 0 to 10 scale, with higher scores indicating greater impact. Two of the items refer to personal health problems and, as the present study focuses on the impact of chronic disease, these items were considered separately in certain analyses.

### Data analyses

The prevalence of depressive symptoms (PHQ-9 scores ≥ 5) was calculated in participants (aged 15 and above) who reported chronic conditions. Student’s *t*-test was used for comparing continuous variables between men and women, while categorical data were analyzed using the *χ*^2^ test. As the depression scores clustered towards the lower end, Spearman ρ correlations were used for bivariate associations of depressive symptoms with potential predictor variables [31]. Variables that were significantly associated with depressive symptoms in the bivariate analyses were then included in multilevel regression models. Multilevel regression was used to analyze predictors as more than one person in each family was interviewed and thus these results would not offer independent observations of the impact of environmental predictor variables. Both beta and standardized beta coefficients were reported to standardize the scales of different psychological assessments in regression analyses. To explore a possible differential effect of social support for men and women, the interaction of family satisfaction and gender was fitted into a multilevel regression model that also included all other significant variables from the bivariate analyses. In addition, the relationship between depressive symptoms and family satisfaction was evaluated separately for men and women using the multilevel regression analysis. For all analyses, two-tailed *p*-values < 0.05 were considered statistically significant. All statistical analyses were performed using IBM-SPSS Statistics software, version 19.0.

## Results

### Study sample profile and prevalence of depressive symptoms

**Figure 1:**
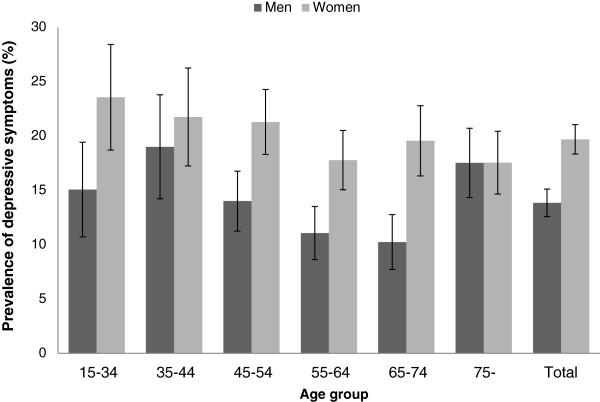
Prevalence of depressive symptoms (PHQ-9 ≥ 5) and their 95% confidence intervals (bar) by age and gender in a Hong Kong population-based sample (N = 6,195) reporting one or more chronic conditions.

A total of 18,907 respondents from 8,481 households completed the interviews. Because of the requirement that all members of a household had to participate, this represented only 22.2% of the households originally approached. Of the 18,907 in the complete sample, 33% (N = 6,239) reported having one or more chronic conditions. A total of 6,195 participants (2,862 men and 3,333 women) had complete data for the present analysis; 44 were excluded due to incomplete responses on the psychological measures. Among them, 17% reported depressive symptoms of mild or higher levels (PHQ-9 score ≥ 5; 13.9% of men and 19.7% of women, *χ*^2^ = 14.4, *p* <0.001). Below age 75, women had a higher prevalence of depressive symptoms than men, but there was no gender difference beyond 75 years (Figure 1). The 3 somatic items in the depression scale contributed substantially to the overall score in this sample of people with a chronic condition, contributing 58% of the score despite forming only one-third of the items (Table 1). The gender difference in response applied almost equally to the somatic (PHQ-3) and non-somatic (PHQ-6) depression scores with a female to male score ratio of 1.38 versus 1.29, respectively (Table 1). Women were less likely than men to be current smokers or problem drinkers; they were more likely to report stressful life events and lower neighborhood cohesion, but reported higher family satisfaction.

**Table 1:** **Characteristics of the study sample with chronic conditions and its difference between men and women**^**a**^

**Variables**	**Total**	**Men**	**Women**	**P values**
Number (%)	6,195 (100%)	2,862 (46.2%)	3,333 (53.8%)	-
Mean age in years (SD) ^b^	58.5 (16.7)	58.4 (16.8)	58.6 (16.6)	0.75
Marital status (%)				<0.001
Never Married	13.0	13.9	12.3	
Married	70.6	78.3	64.0	
Widowed	12.1	4.3	18.9	
Divorced/Separated	4.2	3.5	4.9	
Education levels (%)				<0.001
Primary	44.8	37.4	51.1	
Secondary	39.6	43.2	36.5	
Tertiary or above	15.7	19.4	12.4	
Monthly family income (HK$ ^c^, %)			0.09	
No income or <5,000	26.9	25.3	28.2	
5,000–9,999	17.6	17.2	17.6	
10,000–19,999	25.1	26.1	24.1	
20,000–29,999	14.6	15.1	14.2	
≥ 30,000	15.8	16.4	15.4	
Number of chronic conditions ^d^ (%)				<0.05
1	60.8	62.0	59.7	
2	25.7	25.6	25.8	
≥ 3	13.5	12.4	14.5	
Body mass index, kg/m^2^	24.5 (4.2)	24.5 (3.9)	24.5 (4.4)	0.79
Current high risk drinker (yes, %)	4.8	8.3	1.8	<0.001
Current smoker (yes, %)	12.2	22.1	3.6	<0.001
Physically more active (yes, %)	30.2	30.3	30.1	>0.05
Level of depressive symptom severity (PHQ-9 score, %)				<0.001
Minimal depression (scores 0–4)	83.0	86.2	80.3	
Mild (5–9)	12.8	10.7	14.6	
Moderate (10–14)	2.8	1.9	3.6	
Moderately severe (15–19)	1.0	1.1	1.0	
Severe (≥20)	0.4	0.2	0.5	
Depressive symptoms (PHQ-9 score) ^e^	2.29 (3.38)	1.94 (3.10)	2.60 (3.57)	<0.001
Non-somatic depression score (PHQ-6)	0.95 (2.07)	0.82 (1.91)	1.06 (2.20)	<0.001
Somatic depression score (PHQ-3)	1.34 (1.71)	1.11 (1.57)	1.54 (1.81)	<0.001
Life stress ^f^	0.16 (0.36)	0.14 (0.34)	0.17 (0.37)	<0.001
Living alone (yes, %)	14.7	12.6	16.5	<0.001
Family satisfaction ^g^	6.47 (3.49)	6.31 (3.48)	6.60 (3.50)	<0.01
Neighborhood cohesion ^h^	2.49 (0.62)	2.53 (0.60)	2.47 (0.63)	<0.001

^a^ P values for difference between men and women.

^b^ Unless otherwise indicated, data presented are mean (standard division).

^c^ US$ 1 = HK$7.8.

^d^ Summed by self-reported hypertension, heart disease, stroke, diabetes, dyslipidemia, asthma, chronic obstructive lung disease, digestive disease, chronic musculoskeletal condition and cancer.

^e^ Measured by PHQ-9; scores range from 0 to 27, higher scores indicate greater severity of depressive symptom.

^f^ Measured by the stressful life event questionnaire; scores range from 0 to 4.59, higher scores indicate greater life events related stress.

^g^ Measured by Family APGAR; scores range from 0 to 10, higher scores indicate greater satisfaction with family support.

^h^ Perceived neighborhood social cohesion; scores range from 1 to 5, higher scores indicate greater social cohesion.

### Socio-demographic factors associated with reporting depressive symptoms

The Spearman correlations of PHQ-9 depression scores with age, marital status, and neighborhood cohesion were significant but low, ranging from −0.06 to −0.09; correlations with living alone, alcohol problem drinking, physical activity and with body mass index, were all below 0.05. There was no significant correlation between depression and smoking, family monthly income, or education. After adjustment for age and sex, mean PHQ-9 depression scores were positively associated with the number of chronic conditions with mean scores of 2.1, 2.5 and 3.7 for 1, 2 and ≥3 chronic conditions, respectively (*p* <0.001 for increasing trend). Stronger correlations were observed between PHQ-9 scores and life stress (ρ = 0.40), number of chronic conditions (ρ = 0.11), and family satisfaction (ρ = −0.18) (all *p* <0.05).

The multilevel regression model showed that variables associated with PHQ-9 scores at the bivariate level taken together explained 43% of the variance in PHQ-9 depression scores, and 35% of the variance in the PHQ-6 scores (Table 2). Life stress, the number of chronic conditions and satisfaction with family support were found to be the three strongest predictors of both PHQ-9 (standardized coefficients of 0.46, 0.15, and −0.12 respectively, all *p* <0.001) and PHQ-6 scores (standardized coefficients of 0.44, 0.12, and −0.1 respectively, all *p* <0.001). After the adjustment for other variables in the regression analyses, age and living alone did not contribute any variance in PHQ-9 scores, while living alone and problem drinking did not contribute any variance in PHQ-6 scores (see Table 2).

**Table 2:** **The multilevel regression model of depressive symptoms**^**a**^**and PHQ**-**6 scores with demographic variables**^**b**^, **life stress**, **family satisfaction**, **and neighborhood cohesion in those with chronic conditions** (**N** = **6**,**195**)

**Variables**	**PHQ**-**9 scores**				**PHQ**-**6 scores**			
	**Unstandardized coefficients**	**Std**. **Error**	**Standardized coefficients**	**t**	**Unstandardized coefficients**	**Std**. **Error**	**Standardized coefficients**	**t**
Age (years)	0.00 ^c^	0.00	0.02	1.39	0.00 ^c^ **	0.00	0.04	2.84
Gender (female *vs*. male)	0.55***	0.08	0.09	7.31	0.15**	0.05	0.04	3.21
Marital status (unmarried *vs*. married)	0.19*	0.02	0.03	2.06	0.16**	0.06	0.03	2.75
Number of chronic conditions	0.54***	0.04	0.15	11.97	0.27***	0.03	0.12	9.54
Body mass index (kg/m^2^)	−0.05***	0.01	−0.07	−5.21	−0.02**	0.01	−0.04	−3.20
Alcohol problem drinking (yes *vs*. no)	0.36*	0.18	0.03	2.06	0.11	0.11	0.01	1.01
Physical activity (more active *vs*. less active)	−0.23**	0.08	−0.04	−2.76	−0.15*	−0.05	−0.04	−2.91
Life stress	3.84***	0.10	0.46	36.54	2.30***	0.07	0.44	35.0
Family satisfaction	−0.11***	0.01	−0.12	−9.53	−0.05***	0.01	−0.10	−7.61
Gender by family satisfaction interaction	−0.05*	0.02	−0.03	−2.20	−0.03*	0.01	−0.02	−2.25
Neighborhood cohesion	−0.22***	0.06	−0.04	3.47	−0.16***	0.04	0.05	−3.99
Living alone (yes *vs*. no)	0.18	0.12	0.02	1.46	0.09	0.08	0.02	1.19
R^2^ for the full model ^d^								

* p < 0.05; ** p < 0.01; *** p < 0.001.

^a^ Depressive symptoms were measured by the PHQ-9.

^b^ Variables that were significant in bivariate analyses were entered as independent variables in the multilevel regression analysis.

^c^ The coefficients were small and below two decimals.

^d^ Intra-class correlation within family was 0.12 for both PHQ-9 and PHQ-6.

### Impact of life events on the prevalence and severity of depressive symptoms

Acute life stress was positively associated with both prevalence and severity of depressive symptoms (PHQ-9 scores). Among those reporting a stressful life event, 31.1% reported depressive symptoms (PHQ-9 score ≥5), compared to 8.8% in those without a life event (*χ*^2^ = 495.1, *p* <0.001). Among individuals with a PHQ-9 score ≥ 5, 31% of those with a life event reported moderate and higher levels of depressive symptoms (PHQ-9 score ≥10), compared to 12.3% in those without a life event (*χ*^2^ = 47.6, *p* <0.001). In the multilevel regressions, life stress, excluding personal serious health problems, explained 1% of the variance in depressive symptoms (Table 3). Including the two items on health problems as life events explained a further 5% of variance in depressive symptoms for both PHQ-9 and PHQ-6 scores.

**Table 3:** **Multilevel regression analysis to determine the strongest predictors of depressive symptoms in those with chronic conditions** (**N** = **6**,**195**)

**Model** (**PHQ**-**9**)	**Cumulative R**^**2**^	**R**^**2**^**Change**	-**Log**-**likelihood**	**P value of log**-**likelihood Change**
Model 1: Demographic variables ^a^	0.36	0.36	16252	<0.001
Model 2: Demographic factors + all remaining variables ^b^				
Model 3: model 2 + life stress (excluding health problem)	0.37	0.01	15809	<0.001
Model 4: model 2 + life stress ^c^	0.42	0.05	15550	<0.001
Model 5: model 4 + number of chronic conditions	0.43	0.01	15483	<0.001
Model 6: model 4 + family satisfaction	0.43	0.00	15441	<0.001
**Model** (**PHQ**-**6**)				
Model 1: Demographic variables ^a^	0.28	0.28	13268	<0.001
Model 2: Demographic factors + all remaining variables ^b^				
Model 3: model 2 + life stress (excluding health problem)	0.29	0.01	12865	<0.001
Model 4: model 2 + life stress ^c^	0.34	0.05	12625	<0.001
Model 5: model 4 + number of chronic conditions	0.35	0.01	12582	<0.001
Model 6: model 4 + family satisfaction	0.35	0.00	12558	<0.001

^a^ Demographic variables included age, gender, marital status, body mass index.

^b^ All remaining variables included drinking, physical activity, and neighborhood cohesion. In model 2, number of chronic conditions was selected secondly into the model and family satisfaction the third. In next steps, neighborhood cohesion, physical activity, and drinking were also selected but with negligible R^2^ Changes (all < 0.002), which were not presented.

^c^ The 19-item life stress scale, which includes items on health problems, replaced the 17-item scale in the model, explaining 5% more variance in depressive symptom scores compared with the 17-item scale.

### Satisfaction with family support as a mitigating factor for depressive symptoms

Satisfaction with family support was a significant contributor to the variance in depressive symptoms by buffering the adverse impact of life stress and number of chronic conditions in this sample (Table 2). Thus, 37.4% of those reporting a life event but low family satisfaction scored 5 or above on the PHQ-9, compared to 24.4% in those with a life event and high family satisfaction. Further, there was an interaction effect of gender and family satisfaction on both PHQ-9 and PHQ-6 scores (Table 2). After stratifying by gender in the multilevel regression model, the regression coefficient for satisfaction with family support in predicting PHQ-9 score was significantly greater for women than for men (−0.02 *vs*. -0.01, *t* = −2.15, *p* <0.05). This result supported our hypothesis that, among participants with chronic conditions, satisfaction with family relationships was more important in accounting for depressive symptoms in women than men. However, there was no gender difference in the impact of family support on the non-somatic (PHQ-6) depression score.

## Discussion

In the present study, we examined the prevalence and risk factors for self-reported depressive symptoms in a large community sample of individuals with chronic conditions in Hong Kong. We found that 17% of people with chronic conditions living in the community reported depressive symptoms of mild or higher levels (PHQ-9 score ≥ 5). However, the majority of PHQ-9 scores were attributable to the 3 somatic symptoms (item 3, 4 and 5). Our second general finding is that the conventional risk factors identified in Western cultures, such as high risk drinking, lack of physical activity, and being unmarried, were independently but only weakly associated with depressive symptoms in both the PHQ-9 and PHQ-6 scores in this sample. The third general finding is that the somatic items (3, 4 and 5) raised the depression score, but did not alter the pattern of predictors. The fourth main finding was that the experience of a stressful life event not only increased the likelihood of reporting depressive symptoms in general, but also increased the severity of depression among those who were depressed (PHQ-9 score ≥5). Lastly, satisfaction with family relationships, consistent with our hypotheses, was protective against depression, particularly for women.

A recent study using a community sample of 1,433 elderly (aged 65 and above) Chinese in Hong Kong reported that depression is independently associated with six medically unexplained somatic symptoms [29]. Although that study included both genders (72.8% female), no information on gender difference was available [29]. Our results are consistent with the published literature that women are more likely to suffer from depressive symptoms than men in China [9]. We further indicate that the gender difference in reporting depressive symptoms was only slightly stronger for the somatic symptoms with a female to male ratio of 1.38, compared to 1.29 for the non-somatic items in this sample of people with chronic physical conditions. Our finding suggests that gender, compared with cultural perspective, might have less impact on somatic symptoms in the Chinese populations. The finding of a weak, although significant, negative association of body mass index and physical activity with depressive symptoms is also consistent with the literature [37,47,48]. It has been proposed that physical activity may divert negative thoughts and also elevate mood by enhancing secretion of endorphins [49], but this does not appear to be the case in this sample. Overall, demographic and lifestyle factors contributed to about one third of the variance of depressive symptoms in this sample.

Life stress and the number of chronic conditions emerged as the most important risk factors for depressive symptoms. Although associations between specific chronic conditions and depressive symptoms have been shown in other Chinese samples, our results have added to the understanding of factors moderating depressive symptoms, including life events and family support. The two items on personal health problems in the 19-item life stress questionnaire explained a unique variance (5%) in depressive symptoms, suggesting the diagnosis of a chronic condition itself formed a significant life event. Social support can be measured in various ways and we examined satisfaction with both family and neighborhood support. The overall impact on reducing depressive symptoms of the two sources of support was comparable (β -0.01 *vs*. -0.02, *z* score 1.27, *p* > 0.05). However, satisfaction with family relationships formed the stronger protective factor for depressive symptoms among women, compared to men. We lacked the data required to explore in more detail the influence of sources of support or stress, such as at work, on the development of depressive symptoms. Our study has identified the characteristics of those with chronic illness who are particularly prone to developing depressive symptoms. For instance, a woman experiencing a stressful life event but lacking family support would be at higher risk. This holds some clinical relevance: it may be valuable for general practitioners to include a brief assessment of family relationships and stresses. Referral for counseling may be warranted where family satisfaction is poor, and promotion of family relationships may be a useful strategy to decrease depression in this high-risk population.

The study’s strengths include its large sample of households randomly selected from all 18 districts in Hong Kong. Second, the study focused on the possible protective role of satisfaction with family relationships for depressive symptoms in a culture that emphasizes the importance of family ties. There are many cultural similarities between Hong Kong and large, rapidly modernizing urban areas in China and other Asian nations; findings in Hong Kong may also be applicable across Asia and perhaps also among Asian immigrants in the Western countries. Third, comparing the risk factors for somatic and non-somatic (PHQ-6) depressive symptoms offered some improvement over previous studies. Somatic symptoms may potentially confound the relationship between reporting of chronic conditions and depression, and thus analyzing somatic symptoms separately attempts to address this. The use of chronic conditions with mostly objective clinical endpoints in this study reduces the risk of somatization leading to the reporting of chronic conditions. On the other hand, chronic conditions could lead to somatic symptoms in the PHQ-9 such as poor appetite and feeling tired. Thus, inclusion of the somatic symptoms in the PHQ-9 could lead to over-diagnosis of depression in this population. While omitting somatic symptoms in the PHQ-6 may lead to under diagnosis of depression, especially in a cultural setting where somatization is a relatively common manifestation of depression. Balancing these potential errors is a common diagnostic challenge for the clinician. The study has three main limitations. First, the approach of including only complete households lowered the response rate and could result in selection bias towards participation by more cohesive families with supportive relationships. We tested this possibility by comparing family satisfaction scores for the sample of complete households with an additional sample that included respondents from households in which not all members participated. Family satisfaction was only slightly different: mean of family APGAR 6.5 *vs*. 7.0, effect size = 0.145. Furthermore, the demographic characteristics of the whole sample were similar to the Hong Kong census [32]. The second limitation is that, without knowledge of disease duration and without blood sample collection, we could not adequately assess the severity of the chronic conditions recorded in the sample and the impact of disease control on the associations of depressive symptoms with chronic conditions. Third, the interpretation of our findings was limited by the cross-sectional design. The second wave of the FAMILY project is due to be completed by May 2013, and the prospective data should enable us to more fully explore the interplay of life events, perceived family support and chronic conditions in development of depression, as well as examining the temporal relationship between family support and depression.

## Implications and conclusions

Successful management of long-term health requires the patient’s active participation in following the clinician's recommendations and in making appropriate changes to health behaviors. Depression can inhibit the patient’s participation in the care plan, so it becomes clinically relevant to anticipate when a patient with a chronic condition may develop secondary depression. This study identified readily measurable characteristics of people with chronic illness who are prone to developing depressive symptoms: crucially, their level of stress and their family support. From this, general practitioners could apply a brief prognostic assessment. Although this Chinese community sample did not identify behaviors such as alcohol consumption or lack of exercise as predictors of depression, the weight of previous findings suggests that general practitioners should also consider addressing these after diagnosing a patient with a chronic condition as an approach to maintaining mental well-being. Where an elevated risk of depression is found, referral for counseling may be warranted where family satisfaction is poor, and promotion of family relationships may be a useful strategy to decrease depression in this high-risk population.

## Appendix 1

Patients Health Questionnaire (PHQ-9)

Over the last 2 weeks, how often have you been bothered by any of the following problems?

^a^ Items 3–5 are classified as somatic symptoms

**Table:** 

**Read each item carefully**, **and circle your response**		**Not at all**	**Several days**	**More than half of the day**	**Nearly every day**
1	Little interest or pleasure in doing things	0	1	2	3
2	Feeling down, depressed, or hopeless	0	1	2	3
3^a^	Trouble falling asleep, staying asleep, or sleeping too much	0	1	2	3
4^a^	Feeling tired or having little energy	0	1	2	3
5^a^	Poor appetite or overeating	0	1	2	3
6	Feeling bad about yourself, feeling that you are a failure, or feeling that you have let yourself or your family down	0	1	2	3
7	Trouble concentrating on things such as reading the newspaper or watching television	0	1	2	3
8	Moving or speaking so slowly that other people could have noticed. Or being so fidgety or restless that you have been moving around a lot more than usual	0	1	2	3
9	Thinking that you would be better off dead or that you want to hurt yourself in some way	0	1	2	3

## Abbreviation

PHQ-9: 9-item Patient Health Questionnaire; PHQ-6: 6-item Patient Health Questionnaire; PHQ-3: 3-item Patient Health Questionnaire.

## Competing interests

The authors declare that they have no competing interests.

## Authors’ contributions

HN analyzed the data, contributed to discussions and drafted and edited the manuscript. PHL analyzed the data and contributed to discussions. IM contributed to discussions, suggestions for analyses and finalization of the manuscript. MN contributed to discussions and editing of the manuscript. SMS guided the conceptualization and drafting of the study. THL as principal investigator was responsible for the design and implementation of the FAMILY project, and for editing and finalizing the paper. All authors read and approved the final manuscript.

## Disclaimer

The sponsor was not involved in data collection, analysis, interpretation, writing of the manuscript, or the decision to summit the paper for publication.

## Pre-publication history

The pre-publication history for this paper can be accessed here:

http://www.biomedcentral.com/1471-244X/12/198/prepub

## References

[B1] Moussavi S Chatterji S Verdes E Tandon A Patel V Ustun B Depression, chronic diseases, and decrements in health: results from the World Health Surveys Lancet 2007 370 9590 851 858 10.1016/S0140-6736(07)61415-9 17826170

[B2] Lee S Ling Y Tsang A Community-based co-morbidity of depression and chronic physical illnesses in Hong Kong Int J Psychiatry Med 2010 40 3 339 348 10.2190/PM.40.3.h 21166342

[B3] Li Y Chen C Tu H Cao W Fan S Ma Y Xu Y Hua Q Prevalence and risk factors for depression in older people in Xi’an China: a community-based study Int J Geriatri Psychiatry 2012 27 1 31 39 10.1002/gps.2685 21284042

[B4] Luppa M Sikorski C Luck T Weyerer S Villringer A Konig HH Riedel-Heller SG Prevalence and risk factors of depressive symptoms in latest life–results of the Leipzig Longitudinal Study of the Aged (LEILA 75+) Int J Geriatr Psychiatry 2012 27 3 286 295 10.1002/gps.2718 21538535

[B5] Kessler RC Birnbaum HG Shahly V Bromet E Hwang I McLaughlin KA Sampson N Andrade LH De Girolamo G Demyttenaere K Age differences in the prevalence and co-morbidity of DSM-IV major depressive episodes: Results from the WHO world mental health survey initiative Depress Anxiety 2010 27 4 351 364 10.1002/da.20634 20037917 PMC3139270

[B6] Asia Pacific Cohort Studies Collaboration Cholesterol, diabetes and major cardiovascular diseases in the Asia-Pacific region Diabetologia 2007 50 11 2289 2297 17909878 10.1007/s00125-007-0801-2

[B7] Gu D Gupta A Muntner P Hu S Duan X Chen J Reynolds RF Whelton PK He J Prevalence of cardiovascular disease risk factor clustering among the adult population of China: results from the International Collaborative Study of Cardiovascular Disease in Asia (InterAsia) Circulation 2005 112 5 658 665 10.1161/CIRCULATIONAHA.104.515072 16043645

[B8] Chu NF Wang DJ Liou SH Shieh SM Relationship between hyperuricemia and other cardiovascular disease risk factors among adult males in Taiwan Eur J Epidemiol 2000 16 1 13 17 10.1023/A:1007654507054 10780337

[B9] Phillips MR Zhang J Shi Q Song Z Ding Z Pang S Li X Zhang Y Wang Z Prevalence, treatment, and associated disability of mental disorders in four provinces in China during 2001–05: an epidemiological survey Lancet 2009 373 9680 2041 2053 10.1016/S0140-6736(09)60660-7 19524780

[B10] Kessler RC Merikangas KR Wang PS Prevalence, comorbidity, and service utilization for mood disorders in the United States at the beginning of the twenty-first century Annu Rev Clin Psychol 2007 3 137 158 10.1146/annurev.clinpsy.3.022806.091444 17716051

[B11] Zhang B Li J Gender and marital status differences in depressive symptoms among elderly adults: The roles of family support and friend support Aging Ment Health 2011 15 7 844 854 10.1080/13607863.2011.569481 21562986

[B12] Dupertuis LL Aldwin CM BossÉ R Does the source of support matter for different health outcomes? J Aging Health 2001 13 4 494 510 10.1177/089826430101300403 11917886

[B13] Chi I Chou KL Social support and depression among elderly Chinese people in Hong Kong Int J Aging Hum Dev 2001 52 3 231 252 10.2190/V5K8-CNMG-G2UP-37QV 11407488

[B14] Cheng ST Chan ACM Filial piety and psychological well-being in well older Chinese J Gerontol B Psychol Sci Soc Sci 2006 61 5 P262 P269 10.1093/geronb/61.5.P262 16960229

[B15] Yu J Li J Cuijpers P Wu S Wu Z Prevalence and correlates of depressive symptoms in Chinese older adults: a population-based study Int J Geriatri Psychiatry 2012 27 3 305 312 10.1002/gps.2721 21538538

[B16] Leifheit-Limson EC Reid KJ Kasl SV Lin H Jones PG Buchanan DM Parashar S Peterson PN Spertus JA Lichtman JH The role of social support in health status and depressive symptoms after acute myocardial infarction Circ Cardiovasc Qual Outcomes 2010 3 2 143 150 10.1161/CIRCOUTCOMES.109.899815 20160162 PMC3016989

[B17] Choi NG Ha JH Relationship between spouse/partner support and depressive symptoms in older adults: gender difference Aging Ment Health 2011 15 3 307 317 10.1080/13607863.2010.513042 21140305 PMC3608851

[B18] Wong OMH Gender and intimate caregiving for the elderly in Hong Kong J Aging Studies 2005 19 3 375 391 10.1016/j.jaging.2004.07.007

[B19] Chao SF Assessing social support and depressive symptoms in older Chinese adults: a longitudinal perspective Aging Ment Health 2011 15 6 765 774 10.1080/13607863.2011.562182 21838514

[B20] Kawachi I Berkman L Social ties and mental health J Urban Health 2001 78 3 458 467 10.1093/jurban/78.3.458 11564849 PMC3455910

[B21] Monroe SM Modern approaches to conceptualizing and measuring human life stress Annu Rev Clin Psychol 2008 4 33 52 10.1146/annurev.clinpsy.4.022007.141207 17716038

[B22] Kendler KS Kuhn J Prescott CA The interrelationship of neuroticism, sex, and stressful life events in the prediction of episodes of major depression Am J Psychiatry 2004 161 4 631 636 10.1176/appi.ajp.161.4.631 15056508

[B23] Paykel ES Life events and affective disorders Acta Psychiatr Scand 2003 108 61 66 12956817 10.1034/j.1600-0447.108.s418.13.x

[B24] Tao M Li Y Xie D Wang Z Qiu J Wu W Sun J Wang Z Tao D Zhao H Examining the relationship between lifetime stressful life events and the onset of major depression in Chinese women J Affect Disord 2011 135 1–3 95 99 21821294 10.1016/j.jad.2011.06.054 PMC3210899

[B25] Bhugar D Mastrogianni A Globalisation and mental disorders Br J Psychiatry 2004 184 1 10 20 10.1192/bjp.184.1.10 14702222

[B26] Dinh TQ Yamada AM Yee BWK A culturally relevant conceptualization of depression: An empirical examination of the factorial structure of the Vietnamese Depression Scale Int J Soc Psychiatry 2009 55 6 496 505 10.1177/0020764008091675 19592442

[B27] Kleinman A Culture and depression N Engl J Med 2004 351 10 951 953 10.1056/NEJMp048078 15342799

[B28] Parker G Gladstone G Chee KT Depression in the planet’s largest ethnic group: the Chinese Am J Psychiatry 2001 158 6 857 864 10.1176/appi.ajp.158.6.857 11384889

[B29] Yu DSF Lee DTF Do medically unexplained somatic symptoms predict depression in older Chinese? Int J Geriatri Psychiatry 2012 27 2 119 126 10.1002/gps.2692 22223144

[B30] Spitzer RL Kroenke K Williams JB Validation and utility of a self-report version of PRIME-MD: the PHQ primary care study JAMA 1999 282 18 1737 1744 10.1001/jama.282.18.1737 10568646

[B31] Kroenke K Spitzer RL Williams JB The PHQ-9: validity of a brief depression severity measure J Gen Intern Med 2001 16 9 606 613 10.1046/j.1525-1497.2001.016009606.x 11556941 PMC1495268

[B32] Yu X Tam WW Wong PT Lam TH Stewart SM The Patient Health Questionnaire-9 for measuring depressive symptoms among the general population in Hong Kong Compr Psychiatry 2012 53 1 95 102 10.1016/j.comppsych.2010.11.002 21193179

[B33] Jorm AF Does old age reduce the risk of anxiety and depression? A review of epidemiological studies across the adult life span Psychol Med 2000 30 1 11 22 10.1017/S0033291799001452 10722172

[B34] Cheng ST Chan ACM Social support and self-rated health revisited: is there a gender difference in later life? Soc Sci Med 2006 63 1 118 122 10.1016/j.socscimed.2005.12.004 16443314

[B35] Golden SH Williams JE Ford DE Yeh HC Paton Sanford C Nieto FJ Brancati FL Depressive symptoms and the risk of type 2 diabetes: the Atherosclerosis risk in communities study Diabetes Care 2004 27 2 429 435 10.2337/diacare.27.2.429 14747224

[B36] Boden JM Fergusson DM Alcohol and depression Addiction 2011 106 5 906 914 10.1111/j.1360-0443.2010.03351.x 21382111

[B37] Paluska SA Schwenk TL Physical activity and mental health: current concepts Sports Med 2000 29 3 167 180 10.2165/00007256-200029030-00003 10739267

[B38] Mead GE Morley W Campbell P Greig CA McMurdo M Lawlor DA Exercise for depression Cochrane Database Syst Rev 2009 3 CD004366 19588354 10.1002/14651858.CD004366.pub4

[B39] Cassano P Fava M Depression and public health: an overview J Psychosom Res 2002 53 4 849 857 10.1016/S0022-3999(02)00304-5 12377293

[B40] Guidelines for Data Processing and Analysis of the International Physical Activity Questionnaire (IPAQ) - Short Form, Version 2.0 http://www.institutferran.org/documentos/Scoring_short_ipaq_april04.pdf

[B41] Chan SSC Viswanath K Au DWH Ma CMS Lam WWT Fielding R Leung GM Lam T-H Hong Kong Chinese community leaders’ perspectives on family health, happiness and harmony: a qualitative study Health Educ Res 2011 26 4 664 674 10.1093/her/cyr026 21536713

[B42] Smilkstein G The family APGAR: a proposal for a family function test and its use by physicians J Fam Pract 1978 6 6 1231 1239 660126

[B43] Chan DH Ho SC Donnan SPB A survey of family APGAR in Shatin private ownership homes HK Pract 1988 10 7 3295 3299

[B44] Sampson RJ Raudenbush SW Earls F Neighborhoods and violent crime: a multilevel study of collective efficacy Science 1997 277 5328 918 924 10.1126/science.277.5328.918 9252316

[B45] Mujahid MS Diez Roux AV Morenoff JD Raghunathan T Assessing the measurement properties of neighborhood scales: from psychometrics to ecometrics Am J Epidemiol 2007 165 8 858 867 10.1093/aje/kwm040 17329713

[B46] Miller MA Rahe RH Life changes scaling for the 1990s J Psychosom Res 1997 43 3 279 292 10.1016/S0022-3999(97)00118-9 9304554

[B47] Goodwin RD Association between physical activity and mental disorders among adults in the United States Prev Med 2003 36 6 698 703 10.1016/S0091-7435(03)00042-2 12744913

[B48] Zhi BL Ho SY Chan WM Ho KS Li MP Leung GM Lam HT Obesity and depressive symptoms in Chinese elderly Int J Geriatr Psychiatry 2004 19 1 68 74 10.1002/gps.1040 14716701

[B49] Hegadoren KM O’Donnell T Lanius R Coupland NJ Lacaze-Masmonteil N The role of [beta]-endorphin in the pathophysiology of major depression Neuropeptides 2009 43 5 341 353 10.1016/j.npep.2009.06.004 19647870

